# A randomized controlled trial on the efficacy of life review therapy targeting incurably ill cancer patients: do their informal caregivers benefit?

**DOI:** 10.1007/s00520-020-05592-w

**Published:** 2020-07-03

**Authors:** Gitta Kleijn, Birgit I. Lissenberg-Witte, Ernst T. Bohlmeijer, Vincent Willemsen, Annemarie Becker-Commissaris, Corien M. Eeltink, Anna M.E. Bruynzeel, Maurice J. van der Vorst, Pim Cuijpers, Irma M. Verdonck-de Leeuw

**Affiliations:** 1 Department of Clinical, Neuro- & Developmental Psychology, Vrije Universiteit Amsterdam, Amsterdam, Netherlands; 2 Cancer Center Amsterdam, Amsterdam, Netherlands; 3 Department of Epidemiology and Biostatistics, Amsterdam UMC, location VUmc, Amsterdam, Netherlands; 4 Department of Psychology, Health & Technology, University Twente, Enschede, Netherlands; 5 Center for Psychosocial Oncology Care, Ingeborg Douwes Center, Amsterdam, Netherlands; 6 Department of Pulmonary Diseases, Amsterdam UMC, location VUmc, Amsterdam, Netherlands; 7 Department of Haematology, Amsterdam UMC, location VUmc, Amsterdam, Netherlands; 8 Department of Radiation Oncology, Amsterdam UMC, location VUmc, Amsterdam, Netherlands; 9 Department of Medical Oncology, Amsterdam UMC, location VUmc, Amsterdam, Netherlands; 10 Department of Otolaryngology/Head and Neck Surgery, Amsterdam UMC, location VUmc, Amsterdam, Netherlands

**Keywords:** Cancer, Caregiver burden, Distress, Life review therapy, Palliative care, Partners

## Abstract

**Purpose:**

Investigate whether Life Review Therapy and Memory Specificity Training (LRT-MST) targeting incurably ill cancer patients may also have a beneficial effect on caregiving burden, symptoms of anxiety and depression, and posttraumatic growth of the informal caregivers.

**Methods:**

Data was collected in the context of a randomized controlled trial (RCT) (secondary analyses) on the effect of LRT-MST among incurably cancer patients. Informal caregivers of participating patients were asked to complete outcome measures at baseline (T0), post-intervention (T1), and 1-month follow-up (T2): caregiver burden (caregivers reaction assessment scale (CRA)), symptoms of anxiety and depression (hospital anxiety and depression scale), and posttraumatic growth (posttraumatic growth inventory). Linear mixed models (intention to treat) were used to assess group differences in changes over time. Effect size and independent samples *t* tests were used to assess group differences at T1 and T2.

**Results:**

In total, 64 caregivers participated. At baseline, 56% of the caregivers experienced anxiety and 30% depression. No significant effect was found on these symptoms nor on posttraumatic growth or most aspects of caregiver burden. There was a significant effect of LRT-MST on the course of self-esteem (subscale CRA) (*p* = 0.013). Effect size was moderate post-intervention (ES = − 0.38, *p* = 0.23) and at 3-month follow-up (ES = 0.53, *p* = 0.083).

**Conclusions:**

Many caregivers of incurably ill cancer patients experience symptoms of anxiety and depression. LRT-MST does not improve symptoms of depression and anxiety, negative aspects of caregiver burden, or posttraumatic growth. LRT-MST may have a protective effect on self-esteem of informal caregivers (positive aspect of caregiver burden).

**Trial registration number:**

Netherlands Trial Register (NTR 2256), registered on 23-3-2010.

**Electronic supplementary material:**

The online version of this article (10.1007/s00520-020-05592-w) contains supplementary material, which is available to authorized users.

## Background

Informal caregivers of incurably ill cancer patients face a broad variety of tasks, assisting the patient with disease and treatment monitoring, symptom management, medication, personal and instrumental care, and financial and emotional support [1–3]. Often, caregivers take these responsibilities with little or no preparation or training and with limited resources [2], and they feel committed to provide limitless care [2, 4]. Pitceathly and Maguire [5] showed in a systematic review that most informal caregivers cope well, but also that part becomes distressed or develops mental health problems. Their review showed that, based on self-report questionnaires, 20–30% of caregivers are at risk for psychiatric morbidity. In caregivers of patients with advanced cancer in palliative care, this rate is 30–50%. In studies using diagnostic interviews, lower levels of morbidity were found, ranging from 10% among carers of newly diagnosed patients to 33% among carers of terminally ill patients [5]. Rha et al. [2] reported that family caregivers experience a considerable amount of distress in their efforts to provide care for a cancer patient. This happens especially if the demand of care exceeds the resources of the caregiver, which causes the caregiver to feel overwhelmed and experiences a high level of stress. This stress negatively affects psychological well-being but can also negatively affect physical well-being [6]. The negative effects of caregiving on psychological well-being include increased emotional distress, anxiety, and/or depression (with rates up to 40% in case of palliative care), feelings of helplessness, loss of control, signs of posttraumatic stress disorder, uncertainty, and hopelessness [6, 7]. Despite these challenges, being a caregiver can also result in experiencing positive psychological changes, like personal growth and psychological strength [8]. In a randomized controlled trial (RCT) [9], we examined the efficacy of a life-review intervention named Dear Memories [10, 11], which combines life review therapy with memory specificity training (LRT-MST), among incurably ill cancer patients. We found a positive effect on ego integrity. Ego integrity is described as accepting your life cycle as something that had to be, feeling connected to others, and experiencing a sense of wholeness, meaning, and coherence when facing death) [9]. Additionally, reasons to start, experiences, and perceived outcomes were studied via a qualitative approach among patients who underwent LRT-MST [12]. Patients reported positive outcomes on ego integrity and psychological well-being in the here and now, as well as in the nearby future (including end of life). Also, patients noted that they appreciated sharing and regaining memories and some noted positive outcomes on their social life, e.g., increased social interaction, enjoying having people around again.

Two meta-analyses [13, 14] evaluating emotional distress in cancer patients and their informal caregivers reported that distress was correlated and that couples often react as an “emotional system” [6]. Therefore, in the present study, we investigated whether LRT-MST offered to incurably ill cancer patients (but not to the informal caregivers themselves) may also have an effect on their informal caregivers. The social, instrumental, and integrative functions of reminiscence and life review may lead to improved psychological resources (such as social support, mastery, coping, meaning in life, and self-esteem), which may lead to improved mental health and well-being (such as less depressive and anxiety feelings, and more happiness and life satisfaction [15]. The results are relevant, because previous research showed that caregivers are less likely to disclose their own concerns and worries as their primary focus is on the patients’ need and often they do not to seek help [5].

## Methods

### Study design and population

This study was conducted (June 2010 until December 2013) in the context of a randomized controlled trial (RCT) (secondary analyses) evaluating the efficacy of LRT-MST targeting cancer patients in palliative care [9]. The inclusion criterium was being an informal caregiver of a patient participating in this RCT. Of the 107 (55 randomized in the intervention group; 52 in the waiting list control group) incurably ill cancer patients (all types of cancer and all palliative care modalities), 75 had an informal caregiver (70%), who were asked to participate (the intervention was not targeting the informal caregivers themselves, but the patients only). In total, 64 caregivers provided informed consent (85%). Reasons for not participating are unknown. These 64 caregivers were asked to complete questionnaires (at home) at the same assessment times as the patients: before the start of the intervention (baseline; T0), after the intervention or after 4-week waiting time in the control group (post-treatment; T1), and at 1-month post-treatment (follow-up; T2). Caregivers who participated in the current study followed treatment allocation of the patients in the RCT [9]. The RCT was approved by the Medical Ethics Committee of VU University Medical Center and registered in the Netherlands Trial Register (NTR 2256).

### Intervention

LRT-MST called “Dear Memories” [10] aims to improve the life review process and to train the autobiographical memory, with a focus to retrieve positive specific events from the past. This protocol is based on the life review protocol designed by Serrano et al. [11] for older adults with depressive symptomatology. LRT-MST consists of four weekly sessions covering a particular lifetime period: childhood, adolescence, adulthood, and whole life span. For each period, 14 questions are designed to prompt specific positive memories. Participants are explicitly encouraged to retrieve positive specific memories to the positively stated questions. Each interview, conducted in Dutch, takes approximately 1 h and is led by a psychologist who was trained in the LRT-MST-protocol “Dear Memories.” The intervention takes place at the patient’s residence or at the hospital. The interviews are recorded on mp3, and copies are offered as a remembrance for the patients and/or their informal caregivers [9].

### Care as usual

All informal caregivers (both in the intervention and control group) received care as usual (CAU) which entails physicians and nurses provide emotional support and advice how to cope with being an informal caregiver of an incurably ill cancer patient on an ad hoc basis during hospital visits. Caregivers can also be referred to other services, like a social worker, a psychologist, or the general practitioner.

### Outcome measures

Caregivers completed questionnaires on caregiver burden (caregivers reaction assessment scale; CRA); psychological distress, anxiety, and depressive symptoms (hospital anxiety and depression scale; HADS); and posttraumatic growth (posttraumatic growth inventory; PTGI). Caregivers also filled out a study specific questionnaire on age, gender, relationship status, children, and education level.

The CRA-D (Dutch version) [16, 17] is a 24-item instrument designed to assess subjective caregiver burden and comprises 5 subscales, including both positive (“self-esteem”) and negative burden (“disrupted schedule,” “financial problems,” “lack of family support,” and “loss of physical strength”). Answers were rated on a 5-point Likert scale, ranging from 1 to 5. In the present study, Cronbach’s alpha was 0.75 for care-derived self-esteem subscale, 0.79 for “disrupted schedule” subscale, 0.77 for “financial problems” subscale, 0.76 for “lack of family support” subscale, and 0.74 for “health problems” subscale.

The validated Dutch version of the HADS [18] is a 14-item self-assessment scale for measuring psychological distress (HADS-T) and consists of two subscales: anxiety (HADS-A) and depression (HADS-D). Answers are given on a 4-point Likert scale ranging from 0 to 4. The total HADS score ranges from 0 to 42 and the subscales range from 0 to 21. A subscale score from > 7 indicates an increased risk for an anxiety or depressive disorder, and a total score > 14 indicates psychological distress. Cronbach’s alpha in the present study was 0.90, 0.80, and 0.87 for HADS-T, HADS-A, and HADS-D, respectively.

Tedeschi and Calhoun [19, 20] developed the PTGI and defined posttraumatic growth as psychological growth beyond previous levels of functioning, as a result of the struggle with a traumatic event. The PTGI is a 21-item questionnaire measuring posttraumatic growth including five subscales: relating to others, new possibilities, personal strength, spiritual change, and appreciation of life. Answers are given on a 6-point Likert scale with 0 = “I did not experience this change as a result of my crisis” till 5 = “I experienced this change to a very great degree as a result of my crisis.” Subscale scores are calculated via the summation of the given responses to items belonging to the subscale. The total score is derived by the summation of all 21 items and ranges from 0 till 105, and a higher score indicates a higher level of posttraumatic growth. Cronbach’s alpha in the current study for the subscales relating to others, new possibilities, personal strength, spiritual change, and appreciation of life was 0.83, 0.72, 0.85, 0.64, and 0.73 respectively.

### Statistical methods

Descriptive statistics (mean and standard deviation or frequency and percentage) were used to describe the study population characteristics and scores on caregiver burden, psychological distress, and posttraumatic growth. Independent samples *t* test and chi-square test were used to gauge whether randomization resulted in a balanced distribution of caregiver characteristics and outcome measures at baseline across the groups. Intention-to-treat analyses were performed. Changes over time (from baseline to follow-up) between experimental conditions were investigated using linear mixed models with fixed effects for group, assessment, and their two-way interaction and a random intercept for subjects. If changes from baseline to follow-up between groups were significant, an independent samples *t* test was performed to post hoc assess differences between the experimental conditions immediately after the intervention or control period (T1) and follow-up assessment (T2). Effect sizes (ES) were calculated by dividing the difference between the means of the intervention and the waiting list control group at post and follow-up measurements by the standard deviation (SD) of the control group. Low, moderate, and high ES were defined as ES = 0.10–0.30, ES = 0.30–0.50, and ES > 0.50, respectively [21]. For all statistical analyses, a *p* value < 0.05 was considered statistically significant. Analyses were performed with SPSS 24 (IBM Corp., Armonk, NY USA).

## Results

### Study population

**Fig. 1:**
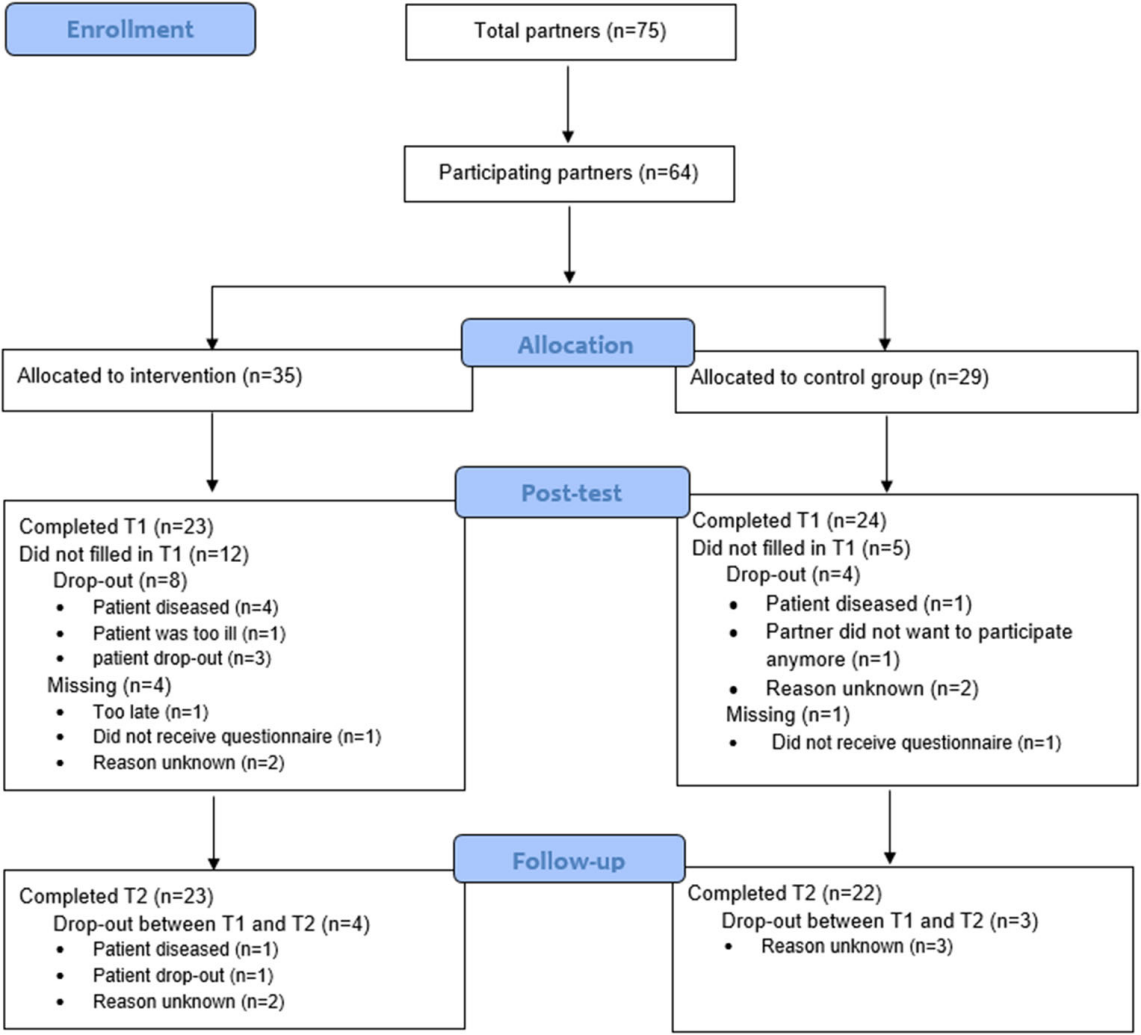
Flow diagram of study population

In total, 64 caregivers participated: 35 caregivers of patients in the LRT-MST condition and 29 caregivers of patients who were randomized in the control group. In total 19 caregivers (30%) did not complete all the questionnaires: 12 in the LRT group and 7 in the control group. Figure 1 shows the flow diagram. An overview of the study population is provided in Table 1. At baseline there were no significant differences between the conditions with respect to sociodemographic characteristics and baseline outcomes. Mean age was 62 years, all except one (who was a brother living with the patient) were partners of the patient, and most were female (61%), had children (88%), and were caregiver of a patient who was treated for lung cancer (63%) or hematological cancer (27%). Many caregivers had an increased risk for an anxiety disorder (56%) or a depressive disorder (30%).

**Table 1:** Overview of patient characteristics

	Total group (*n* = 64)		LRT (*n* = 35)		CAU (*n* = 29)		
	*n*	%	*n*	%	*n*	%	p*
Age							0.093
Mean (SD)	61.6	(9.9)	63.6	(9.2)	59.4	(10.4)	
Range	36–85		50–81		36–85		
Gender							0.73
Male	25	39.1%	13	37.1%	12	41.4%	
Female	39	60.9%	22	62.9%	17	58.6%	
Marital status							0.53
Married	59	92.2%	33	94.3%	26	89.7%	
Not married^	5	7.9%	2	5.7%	3	10.3%	
Children							1.00
No	8	12.5%	4	11.4%	4	13.8%	
Yes	56	87.5%	31	88.6%	25	86.2%	
Level of education							0.48
Primary education	17	27.0%	11	32.4%	6	20.7%	
Secondary education	19	30.2%	8	23.5%	11	37.9%	
Higher education	10	15.9%	6	17.6%	4	13.8%	
Academic education	9	14.3%	6	17.6%	3	10.3%	
Other	8	12.7%	3	8.8%	5	17.2%	
Religion							0.46
No	41	64.1%	21	60.0%	20	69.0%	
Yes	23	35.9%	14	40.0%	9	31.0%	
Tumor type							0.74
Lung cancer	40	62.5%	21	60.0%	19	65.5%	
Hematological cancer	17	26.6%	9	25.7%	8	27.6%	
Breast cancer	1	1.6%	1	2.9%	0	0.0%	
Other	6	9.4%	4	11.4%	2	6.9%	
Risk on psychiatric morbidity							
Anxiety (HADS-A > 7)	36	56.3%	20	58.8%	16	55.2%	0.77
Depression (HADS-D > 7)	19	29.7%	13	38.2%	6	21.4%	0.14

*LRT* intervention group, *CAU* waiting list control group—care as usual, *SD* standard deviation

*Chi-square test (age = independent samples *t* test)

^One couple were brothers living together

### Effect of the intervention on caregivers

Descriptive statistics of the outcome measures at T0, T1, and T2 of the caregivers in the intervention group versus those in the control group are provided in Table 2. A significant change (*p* = 0.013) was found over time on the course of the scores on the subscale “self-esteem” of the CRA: self-esteem of caregivers of patients in the intervention group remained stable over time, while self-esteem in caregivers of patients in the control group decreased. Post hoc analyses showed a moderate ES post-intervention (mean difference = − 0.18, 95% CI = − 0.48–0.12, ES = − 0.38, *t* = − 1.21, *df* = 45, *p* = 0.23) and at 3-month follow-up (mean difference = 0.30, 95% CI = − 0.041–0.64, ES = 0.53, *t* = 1.78, *df* = 43, *p* = 0.083). The results of these post hoc analyses were not statistically significant. No effect was found on the scores of the other subscales of the CRA, HADS, or PTGI.

**Table 2:** Overview of the effect of the intervention on the patient reported outcomes over time

		Pretest			Posttest			Follow-up			Interaction LLM			
		*n*	M	SD	*n*	M	SD	*n*	M	SD	F	df1	df2	*p*
CRA														
Disrupted schedule	LRT	35	3.3	0.8	23	3.2	0.8	23	3.3	0.9	0.07	2	92	0.93
	CAU	28	3.0	1.0	24	2.9	0.9	22	3.0	0.9				
Financial problems	LRT	34	2.5	0.8	23	2.5	0.9	23	2.5	0.8	0.33	2	91	0.72
	CAU	27	2.5	0.9	23	2.8	0.9	22	2.6	1.1				
Lack of family support	LRT	35	2.3	0.6	23	2.3	0.7	23	2.5	0.9	0.31	2	91	0.74
	CAU	28	2.2	0.6	24	2.3	0.8	22	2.3	0.7				
Health problems	LRT	35	2.6	0.7	23	2.6	0.7	23	2.5	0.8	0.23	2	93	0.80
	CAU	28	2.5	0.9	24	2.4	0.7	22	2.5	0.7				
Self-esteem	LRT	35	4.2	0.6	23	4.0	0.5	23	4.1	0.6	4.57	2	94	0.013*
	CAU	28	4.1	0.4	24	4.2	0.5	22	3.8	0.6				
HADS														
Total	LRT	33	14.7	7.7	23	12.3	6.5	23	14.2	7.5	1.27	2	90	0.29
	CAU	28	12.8	5.8	24	14.2	5.8	22	14.8	8.8				
Anxiety	LRT	34	8.3	3.3	23	6.9	3.2	23	7.9	3.5	1.27	2	93	0.29
	CAU	29	7.6	3.4	24	8.0	3.2	22	8.3	4.6				
Depression	LRT	34	6.4	4.6	23	5.5	3.8	23	6.3	4.9	0.92	2	90	0.40
	CAU	28	5.2	3.0	24	6.1	3.4	22	6.5	4.7				
PTGI														
Total	LRT	32	40.3	19.6	21	39.3	19.7	21	41.4	19.	0.62	2	82	0.54
	CAU	26	36.7	21.1	22	37.5	20.6	20	38.4	19.3				
Relating to others	LRT	35	17.1	7.1	22	15.9	7.0	22	16.9	6.7	0.31	2	86	0.73
	CAU	26	14.2	8.2	23	15.3	7.7	20	15.2	8.5				
New possibilities	LRT	33	8.4	5.0	21	8.3	4.8	22	9.5	4.9	0.85	2	93	0.43
	CAU	28	7.3	5.8	24	8.0	5.3	21	7.3	4.7				
Personal strength	LRT	34	7.1	5.9	23	6.5	5.1	23	8.0	4.8	1.38	2	88	0.26
	CAU	28	7.1	5.4	24	7.8	5.7	21	7.6	5.3				
Spiritual change	LRT	35	1.2	2.2	23	1.0	2.2	23	1.1	2.2	0.29	2	92	0.75
	CAU	28	1.1	2.0	24	0.7	1.6	21	1.0	1.6				
Appreciation of life	LRT	34	6.5	4.2	23	6.9	4.0	23	7.1	4.3	0.77	2	89	0.47
	CAU	28	6.2	3.6	23	6.3	3.7	22	6.8	3.7				

*CRA* caregiver reaction assessment, *HADS* hospital anxiety and depression scale, *PTGI* posttraumatic growth inventory, *LRT* intervention group, *CAU* waiting list control group—care as usual, *M* mean, *SD* standard deviation, *F* assessment x group, *p p* value

*p < 0.05

## Discussion

This study showed that LRT-MST targeting patients of incurable ill cancer patients had no significant effect on their caregivers regarding symptoms of depression or anxiety, posttraumatic growth, or most of the subscales of caregiver burden. There was a significant difference on the course of self-esteem (subscale of the CRA) over time. We also found that 56% of the caregivers reported symptoms of anxiety and 30% symptoms of depression at baseline. These percentages are in line with data from a meta-analysis of Pitceathly and Maguire [5]. LRT-MST did not have a significant effect on symptoms of anxiety of depression among caregivers, nor among the patients themselves [9]. In our studies, we did not preselect participants with anxiety of depression, but included all patients and all caregivers. A meta-analysis using individual patient data showed that psychosocial interventions in general seem to be more effective when they target cancer patients with symptoms of anxiety or depression [22], which could explain our findings.

It is striking that LRT-MST also does not seem to be effective on other negative psychological constructs as negative caregiver burden (financial problems, lack of family support, and loss of physical strength) (present study) and despair (among patients [9]), but does seem to have a beneficial effect on positive mental health as self-esteem (among caregivers, present study) and ego integrity (among patients [9]).

Assuming that patients talked about their memories during and after the intervention period with their informal caregivers and the positive (experienced) effect on ego integrity of the patient, it might explain the effect of LRT-MST on self-esteem of caregivers. While self-esteem in caregivers of patients in the control group decreased, self-esteem of caregivers of patients in the intervention group remained stable over time, which suggests that LRT-MST has a protective effect in this group of people who are facing end of life of their loved one. It may be that the social function of reminiscence and life review leads to improved self-esteem [15]. However, positive mental health is complex and involves various theoretical constructs [23]. Previous research showed that data from questionnaires on psychological well-being and personal meaning overlap to a large extent. Posttraumatic growth seemed to be a separate construct, which might explain why no effect was found on posttraumatic growth in the present study. However, the question remains whether this separate construct is an artifact of the different type of item response of the PTGI (which asks about how feelings differ from before cancer instead of how feelings are at the moment). Another question that remains unanswered is whether the intervention itself may trigger participants to respond differently on questionnaires targeting positive mental health compared with questionnaires targeting psychological problems. LRT-MST clearly focusses on retrieving positive memories while actively avoiding negative memories. This may have been of influence on the questionnaire-based data. More qualitative research is needed to understand these complex interrelations and operationalizations of positive and negative psychological constructs and the effect that LRT-MST may or may not have among advanced cancer patients and their informal caregivers.

### Study limitations

This study had some limitations that should be considered. The number of partners included in the study was limited which may have hampered the statistical power of the study and could explain not finding further significant effects. Also, for most partners, item 16 (part of the subscale “disrupted schedule”) of the CRA was missing in the questionnaire by mistake. Therefore, we were unable to make assumptions about this subscale or the total caregiver burden. Also, we do not know the amount of caregiver burden among participants, which may have varied from a couple hours of care per week to many hours per day. Another limitation is the follow-up assessment being only 1 month after treatment, and longer-term effects remain unknown.

### Clinical implications

LRT-MST targeting incurably ill cancer patients may help their informal caregivers to maintain their sense of self-esteem. However, caregivers who suffer from psychological distress (which is common in this population) may be better off with a psychological intervention targeting themselves.

## Conclusions

Many informal caregivers of incurably ill cancer patients experience symptoms of anxiety and depression. LRT-MST targeting incurably ill cancer patients does not seem beneficial for caregivers in reducing symptoms of depression and anxiety, negative aspects of caregiver burden, or facilitating posttraumatic growth. LRT-MST may have a protective effect on self-esteem of informal caregivers (positive aspect of caregiver burden).

## Electronic supplementary material

ESM 1 (SPS 7 kb)

## Data Availability

The authors have full control of the data, and data is available upon request.
